# Nurse-led caregiver training interventions in post-stroke rehabilitation: a systematic review and meta-analysis of functional and psychosocial outcomes

**DOI:** 10.3389/fpubh.2026.1820177

**Published:** 2026-06-03

**Authors:** Sai Gao, Lili Dong, Xia Li

**Affiliations:** 1 The Sixth Ward of the Neurology Department, The First Medical Center of Chinese PLA General Hospital, Beijing, China; 2 Hematology Department, The First Medical Center of Chinese PLA General Hospital, Beijing, China; 3 Department of Rehabilitation Medicine, The First Medical Center of Chinese PLA General Hospital, Beijing, China

**Keywords:** caregiver burden, caregiver training, functional outcomes, nurse-led interventions, post-stroke care, stroke rehabilitation

## Abstract

**Background:**

Stroke survivors often require long-term care from unprepared family caregivers, causing physical, emotional, and social challenges. Nurse-led caregiver training offers education and support to enhance functional recovery, caregiver competence, and psychosocial well-being. This study evaluated whether such interventions improve functional and psychosocial outcomes in stroke survivors and caregivers during post-stroke rehabilitation.

**Methods:**

A systematic review and meta-analysis of eight randomized controlled trials (2015–2025) was conducted using PubMed, Embase, CINAHL, Cochrane Library, and Google Scholar. Data extraction focused on functional and psychosocial outcomes. Statistical analyses were performed with RevMan 5.4 and R, calculating SMD and 95% CI. Heterogeneity was assessed via I^2^, and sensitivity analyses were conducted.

**Results:**

Nurse-led interventions improved stroke survivors’ physical function and motor ability (SMD = 0.60, 95% CI: 0.18–1.02, *p* = 0.005), but not activities of daily living or self-efficacy (SMD = 0.01, 95% CI: −0.29-0.31, *p* = 0.94). Psychosocial outcomes improved, including quality of life (SMD = 0.51, 95% CI: 0.11–0.92, *p* = 0.01) and reductions in anxiety and depression (SMD = 0.49, 95% CI: 0.05–0.93, *p* = 0.03). Caregivers showed significant gains in knowledge/skills (SMD = 1.02, 95% CI: 0.42–1.61, *p* = 0.0008), e-health literacy (SMD = 1.09, 95% CI: 0.55–1.62, *p* < 0.0001), and competence/preparedness (SMD = 0.87, 95% CI: 0.35–1.39, *p* = 0.0001), with no significant improvements in burden, emotional exhaustion, or quality of life. Sensitivity analyses confirmed robustness.

**Conclusion:**

Nurse-led caregiver training improves stroke survivors’ motor and psychosocial outcomes and enhances caregiver knowledge, preparedness, and e-health literacy. However, effects on ADLs and caregiver burden remain uncertain due to low-certainty evidence, supporting integration into rehabilitation while emphasizing the need for stronger future evidence.

**Systematic review registration:**

https://www.crd.york.ac.uk/PROSPERO/view/CRD420251104400, identifier (CRD420251104400).

## Background

Stroke remains a leading cause of disability and death globally, leaving survivors with significant functional and psychosocial challenges. Worldwide, incident strokes affect more than 12 million each year with 7 million deaths and 101 million disability-adjusted life-years lost, with the majority of these affected nations being low- and middle-income earners (1). The transition from hospital to home is particularly vulnerable, as survivors and their caregivers adjust to new roles and rehabilitation regimens. Family caregivers, who provide the bulk of post-stroke care, often experience stress, fatigue, and emotional burden if not adequately supported (2, 3).

Nurse-led caregiver training has emerged as an effective intervention to address these challenges. These programs provide targeted education, practical training, and psychosocial support, empowering caregivers to manage rehabilitation at home, reduce caregiver burden, and enhance family adjustment (4–6). Functional recovery is a key goal of post-stroke rehabilitation. Nurse-led interventions focusing on early mobilization, physical exercises, and self-care activities have demonstrated improvements in activities of daily living, mobility, and independence, while reducing complications such as joint stiffness and muscle atrophy (7, 8). Meta-analytic evidence indicates that nurse-led transitional care programs improve quality of life, functional status, medication adherence, and reduce hospital readmissions compared with standard care (9, 10).

Recent systematic reviews point to fragmented evidence of nurse-led caregiver training in particular. A 2022 Stroke review of 18 dyadic interventions identified reduction of caregiver burden significantly on only 11 studies with variable survivor functional benefits because of the diverse designs and small samples (11). Similarly, a meta-analysis of caregiver-mediated exercises demonstrated that basic ADLs and no effects on extended ADLs, anxiety, and depression were found in survivors (12). Psychosocially, although there is a consistent increase in knowledge, caregiver QoL and burden decreases are still null in 40–50% of trials, especially in the absence of e-health or long-term follow-up (13).

Motor ability improves, but meta-analyses report null ADL/self-efficacy effects, likely due to short interventions and exclusion of pure nurse-led caregiver protocols. No synthesis isolates 2015–2025 RCTs on dyadic outcomes from nurse-led training, excluding multidisciplinary bundles (14, 15).

Beyond functional outcomes, caregivers experience considerable psychosocial strain, including depression, anxiety, and fatigue. Nurse-led interventions offer psychoeducation, emotional support, and coping strategies, improving caregiver satisfaction, self-efficacy, and preparedness (16–18). Telehealth and remote programs, accelerated by the COVID-19 pandemic, facilitate continuous support, skill-building, and monitoring, particularly for caregivers in remote or underserved areas (19, 20). Peer support models and culturally tailored interventions have also been effective, especially in low- and middle-income settings, highlighting the need for equitable and context-specific approaches (21).

Despite the growing evidence, including telehealth-enabled training accelerated by COVID-19, challenges remain, including staffing constraints, variability in program delivery, and a lack of standardized protocols and long-term follow-up measures (3). With the expansion of nurse-led programs and technological support in recent years, a systematic synthesis of their effectiveness is timely and necessary.

This systematic review and meta-analysis (2015–2025) aim to evaluate the effectiveness of nurse-led caregiver training interventions in improving functional recovery and psychosocial outcomes for stroke survivors and their caregivers during post-stroke rehabilitation, providing evidence to guide clinical practice and policy.

## Methods

### Protocol and registration

This systematic review and meta-analysis were conducted in accordance with the PRISMA 2020 guidelines. The protocol was prospectively registered with PROSPERO (CRD420251104400).

### Study design

This study is a systematic review and meta-analysis evaluating the effectiveness of nurse-led caregiver training interventions in post-stroke rehabilitation.

### PICO-criteria

- Population: Adult stroke survivors (≥18 years) and their informal/family caregivers.
- Intervention: Nurse-led caregiver training.
- Comparison: Usual care or non-nurse-led interventions/placebo.
- Outcomes: Functional outcomes (activities of daily living, mobility, functional independence), psychosocial outcomes (caregiver burden, anxiety, depression), and quality of life.
- Study Design: Randomized controlled trials (RCTs).

### Inclusion criteria

- Peer-reviewed quantitative studies (RCTs) evaluating nurse-led caregiver training during post-stroke rehabilitation.
- Adult stroke survivors and informal caregivers.
- Interventions aimed at improving caregiver preparedness and/or patient outcomes.
- Functional and psychosocial outcomes reported.
- Published in English between July 2015 and June 2025.

### Exclusion criteria

- Reviews, case reports, editorials, commentaries, conference abstracts without data, letters, or protocols without results.
- Studies on pediatric stroke (<18 years).
- Non-nurse-led interventions.
- Studies not reporting functional or psychosocial outcomes.
- Studies outside the defined publication period or non-English.

### Search strategy and information sources

Comprehensive searches were performed in Embase, PubMed, CINAHL, the Cochrane Library, and Google Scholar (July 2015–June 2025). Controlled vocabulary and keywords related to stroke, caregiver training, and nurse-led interventions were used (Supplementary Table 1). Additional strategies included contacting study authors for missing data and screening grey literature. Individual patient-level data were not sought; summary data were extracted from published reports.

### Study selection

The selection of studies was conducted using a two-stage screening procedure that was standardized so that each study could be considered to be included in the review or not. All titles and abstracts of records found in the databases were screened by two independent reviewers, and a pre-screening step of duplication removal was done using Rayyan AI. Both reviewers evaluated each of the records and each complete report retrieved, blinding to one another and their decisions. The screening workflow, as well as deduplication, title/abstract screening, and decision logging, was assisted with Rayyan AI.

### Data extraction

Data were extracted using a standardized form including study characteristics, sample demographics, intervention and comparator details, outcomes, and effect sizes. Dyadic data (patient and caregiver) were extracted separately to avoid double-counting. Missing information was clarified by contacting authors.

### Risk of Bias assessment

**Figure 1:**
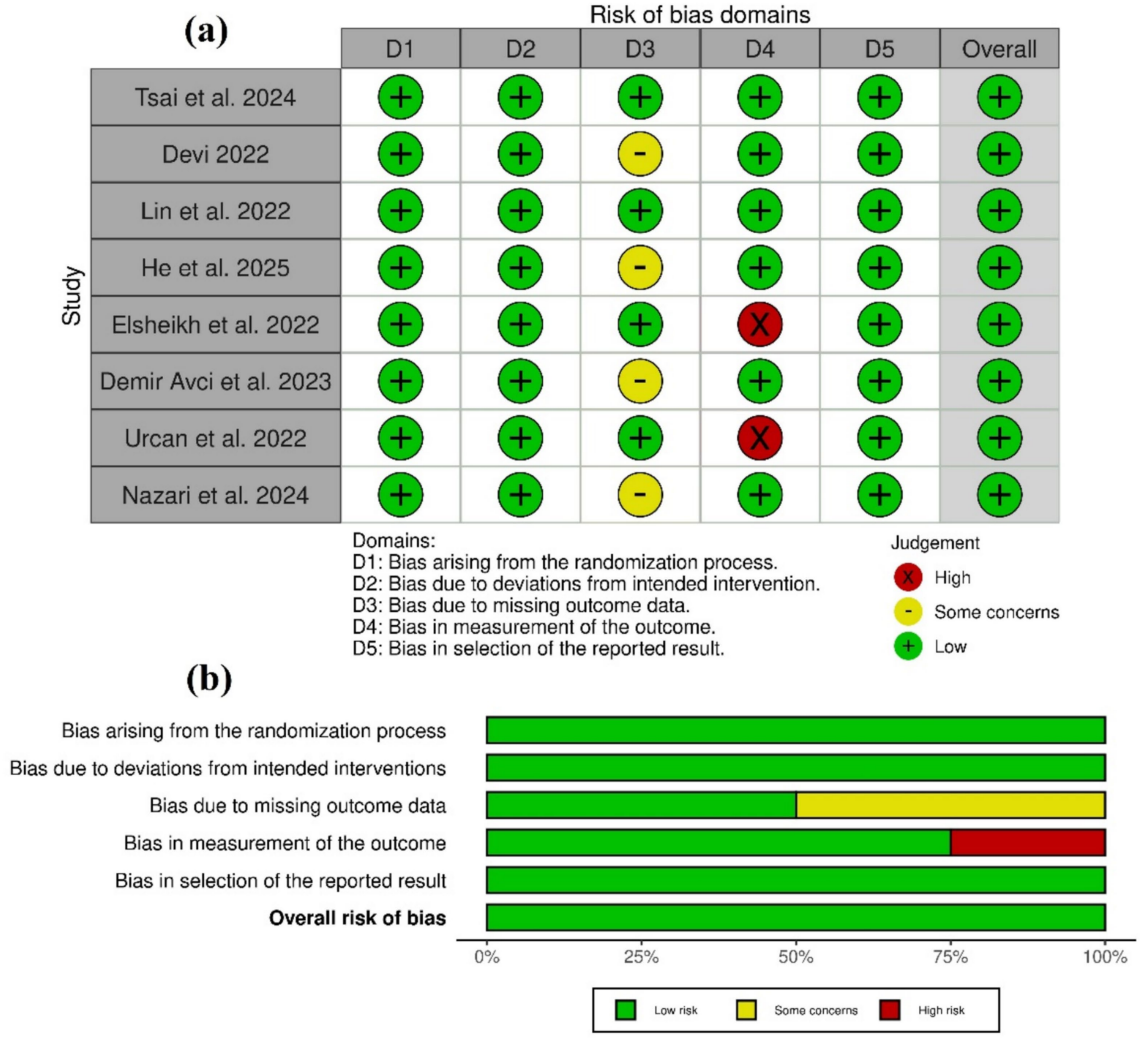
Risk of bias assessment of the included randomized controlled trials (RCTs) using the Cochrane RoB 2.0 tool: **(a)** study-level judgments across five bias domains and overall risk of bias; **(b)** summary of the proportion of studies rated as low risk, some concerns, or high risk in each domain.

The Cochrane Risk of Bias 2.0 tool (22) assessed study quality across key domains. Most studies had low risk in randomization and intervention fidelity, but some concerns were noted for missing outcome data and outcome measurement (Figure 1).

### GRADE evidence quality assessment

The body of evidence of each outcome was evaluated with the GRADE Working Group method to ensure certainty (23). RCTs were originally considered as high-certainty evidence and downgraded on five domains, risk of bias, inconsistency, indirectness, imprecision, and publication bias. The general confidence of each result was classified as high, moderate, low, or very low.

### Statistical analysis

Meta-analysis was conducted using RevMan 5.4 and R. Standardized mean differences (SMD) with 95% confidence intervals (CI) were calculated for continuous outcomes. A random-effects model was used to account for between-study heterogeneity, quantified by I^2^. End-of-treatment data were pooled; multi-arm trials were combined appropriately to avoid unit-of-analysis errors. Sensitivity analyses were performed by excluding studies contributing to heterogeneity.

### Role of the funding source

There was no funding source for this study.

## Results

### Study selection

**Figure 2:**
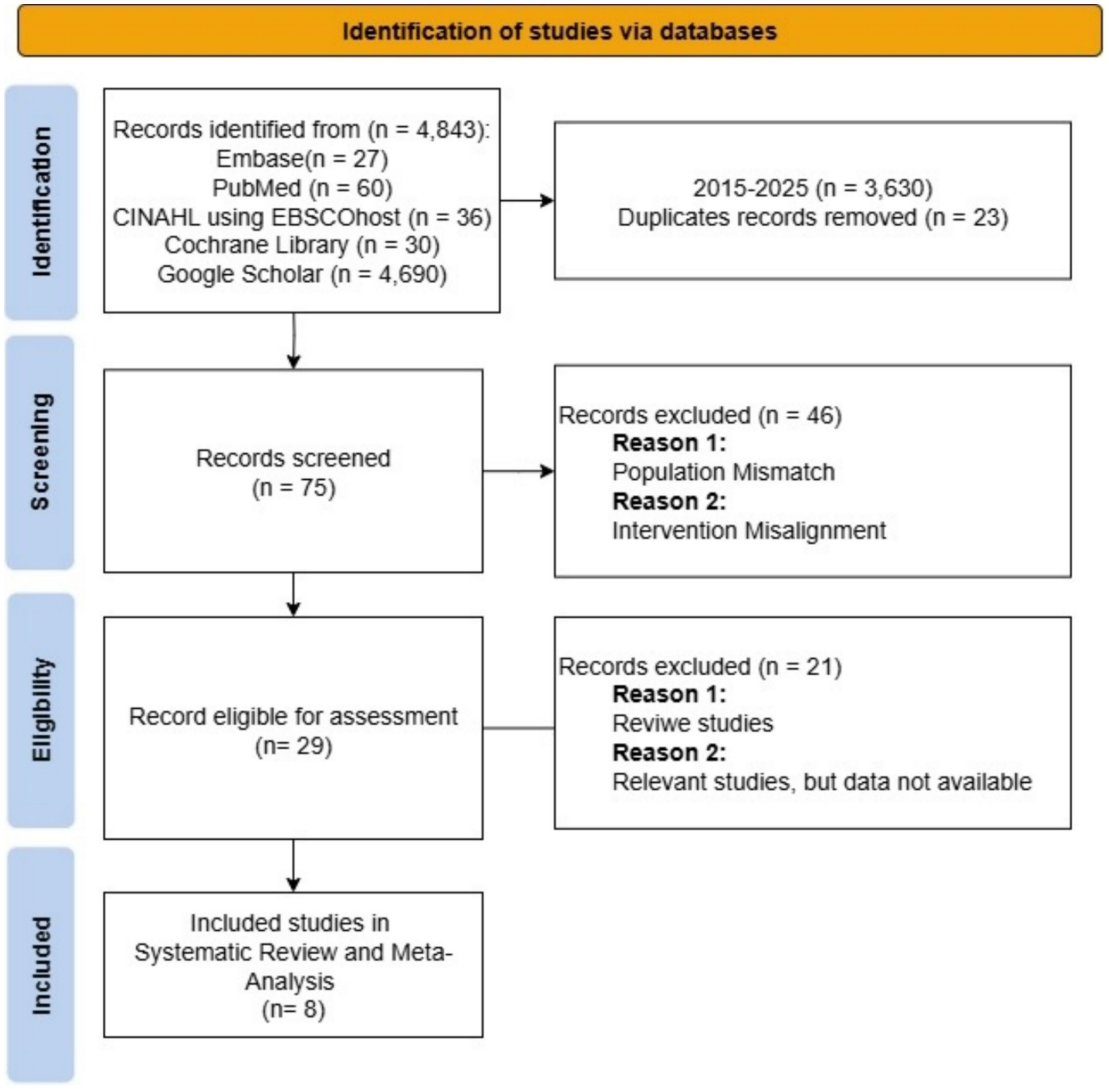
PRISMA 2020 flow diagram showing the study selection process for included RCTs on nurse-led caregiver training interventions in post-stroke rehabilitation.

Figure 2 illustrates the study selection process. Of 4,843 records identified, 3,607 remained after duplicate removal and title/abstract screening. Full-text assessment of 75 records led to exclusion of 67 studies due to population mismatch, intervention misalignment, or insufficient data. Eight RCTs were included in the systematic review and meta-analysis (24–31).

### Study characteristics

The eight included RCTs were conducted across Taiwan, India, China, Egypt, Turkey, and Iran, with sample sizes ranging from 25 to 85 per arm. Intervention durations spanned 3–26 weeks, with follow-up periods of 1–6 months (Table 1). All studies evaluated structured nurse-led training programs targeting functional and psychosocial outcomes in stroke survivors and caregivers.

**Table 1:** Characteristics of Included Studies.

Author’s	Country	Setting	Study design	Sample size IG/CG	Duration	Follow-up
Tsai et al. (2024) (24)	Taiwan	Hospital	RCT	42/40	4 weeks	1 month
Devi (2022) (25)	India	Hospital	RCT	85/85	26 weeks	6 months
Lin et al. (2022) (26)	China	Hospital	RCT	70/70	12 weeks	6 months
He et al. (2025) (27)	China	Rehabilitation	RCT	25/25	3 weeks	3 months
Elsheikh et al. (2022) (28)	Egypt	Community	RCT	55/55	26 weeks	6 months
Demir Avci et al. (2023) (29)	Turkey	Hospital	RCT	66/60	20 weeks	3 months
Urcan et al. (2022) (30)	Turkey	Hospital	RCT	46/46	20 weeks	3 months
Nazari et al. (2024) (31)	Iran	Hospital	RCT	30/30	6 weeks	2 months

### Intervention characteristics

Interventions varied in format (in-person counseling, home visits, health coaching, telehealth, WhatsApp-based programs) and intensity. Nurses acted as educators, facilitators, coordinators, and content developers. Outcomes measured included caregiver burden, patient functional recovery, and quality of life using instruments such as the Barthel Index, Zarit Burden Scale, and Stroke-Specific QoL Scale. Comparators were standard care, often limited to verbal instructions or educational booklets (Table 2).

**Table 2:** Intervention characteristics.

Author’s	Intervention type	Delivery format	Intervention duration	Nurse role	Comparator	Functional outcome measurement tool	Psychosocial outcome measurement tool
Tsai et al. (2024) (24)	Nurse-led therapeutic conversations with STNC-AM	In-person counseling, needs assessments	4 weeks	Lead facilitator, assessment, counseling	Usual care (no structured involvement)	Resilience Scale	General functioning subscale of the McMaster family assessment device (FAD-GF)
Devi (2022) (25)	Nurse-led stroke Education program (NSEP)	Laptop-assisted, follow-up via phone	26 weeks	Lead educator, education and follow-up	Routine care (no stroke education)	Barthel index	Burden Assessment Scale
Lin et al. (2022) (26)	Nurse-led health coaching (goal setting, education, support)	In-person coaching, weekly phone calls	12 weeks	Health coach, educator, coordinator	Usual care (verbal education, follow-up)	Modified caregiver strain index (CSI)	Modified caregiver strain index (CSI)
He et al. (2025) (27)	Video Teach-Back (V&T) health education for caregivers	Video education, teach-back method	3 weeks	Lead educator, guidance, and follow-up	Usual health education	Barthel index (BI)	Questionnaire on stroke knowledge, caregiver skills
Elsheikh et al. (2022) (28)	Tailored multidimensional intervention	Home visits, phone calls, peer support	26 weeks	Educator, facilitator	Educational booklet, one home visit	N/A	Zarit burden interview (ZBI)
Demir Avci et al. (2023) (29)	Transitional care model stroke Turkey (TCM stroke Turkey)	Home visits, phone calls, web-based training	20 weeks	Lead facilitator, education and follow-up	Standard hospital discharge education	Caregiving Competence Scale	Maslach burnout inventory general form (MBI-GF)
Urcan et al. (2022) (30)	Nurse-led education on stroke rehab and sleep quality	Face-to-face sessions, phone follow-up	20 weeks	Educator, coordinator	Standard hospital discharge care	Richards Campbell sleep Questionnaire (RCSQ)	Stroke-Specific Quality Of Life Scale (SSQOL)
Nazari et al. (2024) (31)	Online stroke education via WhatsApp	WhatsApp, audio/video, phone follow-up	6 weeks	Educator, content facilitator	Standard hospital education	N/A	Stroke specific quality of life scale (SS-QOL)

### Functional outcomes in stroke survivors

**Figure 3:**
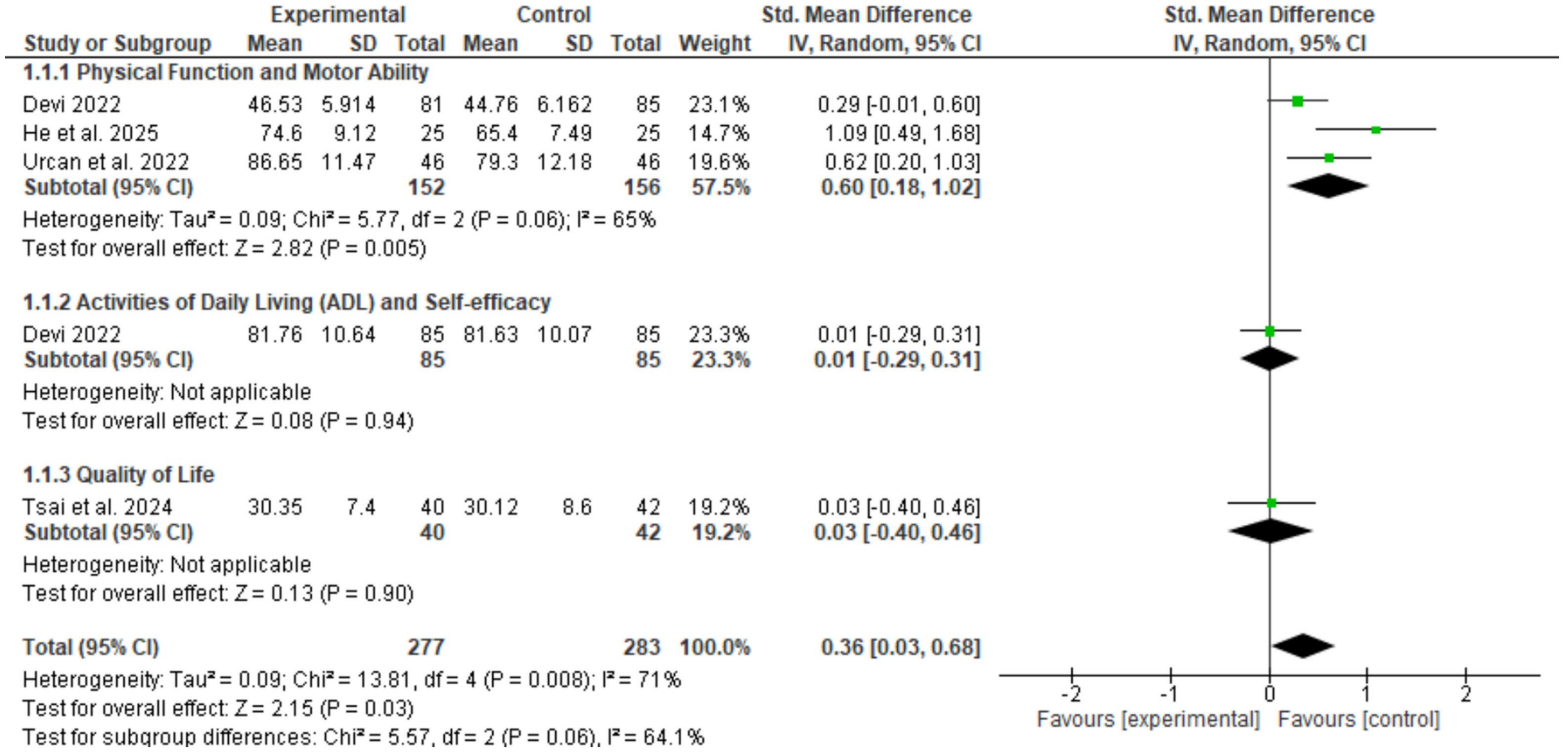
Forest plot of functional outcomes in stroke survivors following nurse-led caregiver training interventions, showing standardized mean differences (SMD) with 95% confidence intervals (CI) for physical function, motor ability, activities of daily living, and self-efficacy.

Meta-analysis showed improvements in physical function and motor ability (SMD = 0.60, 95% CI: 0.18–1.02, *p* = 0.005), but no significant effect on ADLs or self-efficacy (SMD = 0.01, 95% CI: −0.29-0.31, *p* = 0.94). Total pooled effect size was 0.36 (95% CI: 0.03–0.68, *p* = 0.03). Sensitivity analysis reduced heterogeneity from 99 to 71% (Figure 3).

### Functional outcomes in caregivers

**Figure 4:**
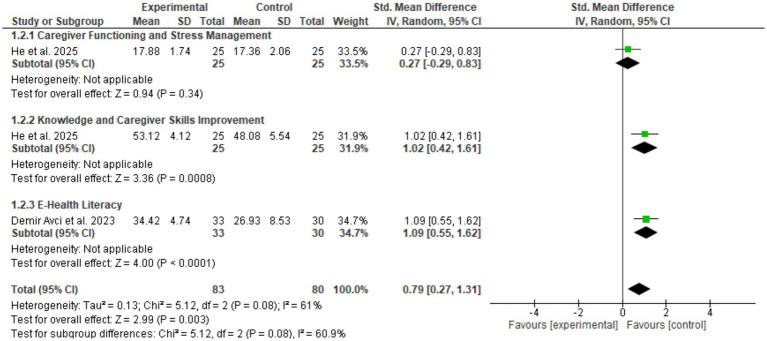
Forest plot of functional outcomes in caregivers following nurse-led caregiver training interventions, showing SMDs with 95% CI for caregiver functioning, stress management, knowledge, skills, and e-health literacy.

No significant effect on caregiver functioning or stress management (SMD = 0.27, 95% CI: −0.29-0.83, *p* = 0.34) was observed, but knowledge and skills (SMD = 1.02, 95% CI: 0.42–1.61, *p* = 0.0008) and e-health literacy (SMD = 1.09, 95% CI: 0.55–1.62, *p* < 0.0001) improved. Total pooled effect size: 0.79 (95% CI: 0.27–1.31, *p* = 0.003). Sensitivity analysis reduced I^2^ from 89 to 61% (Figure 4).

### Psychosocial outcomes in stroke survivors

**Figure 5:**
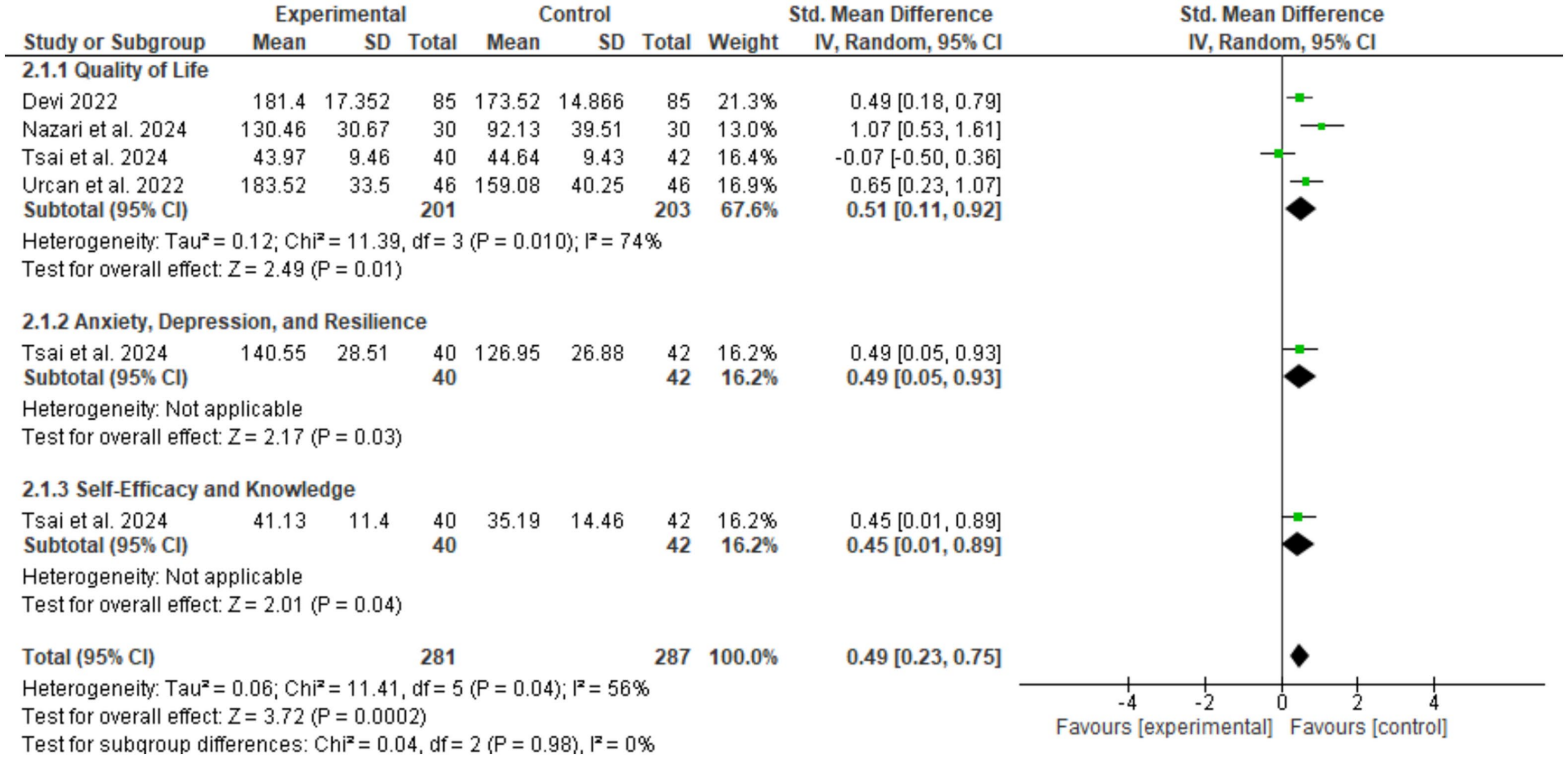
Forest plot of psychosocial outcomes in stroke survivors, showing SMDs with 95% CI for quality of life, anxiety, depression, resilience, self-efficacy, and knowledge following nurse-led caregiver training interventions.

QoL improved moderately (SMD = 0.51, 95% CI: 0.11–0.92, *p* = 0.01), and anxiety/depression decreased (SMD = 0.49, 95% CI: 0.05–0.93, *p* = 0.03). Self-efficacy and knowledge showed smaller improvements (SMD = 0.45, 95% CI: 0.01–0.89, *p* = 0.04). Total pooled effect size: 0.49 (95% CI: 0.23–0.75, *p* = 0.0002). Sensitivity analyses reduced heterogeneity from 98 to 56% (Figure 5).

### Psychosocial outcomes in caregivers

**Figure 6:**
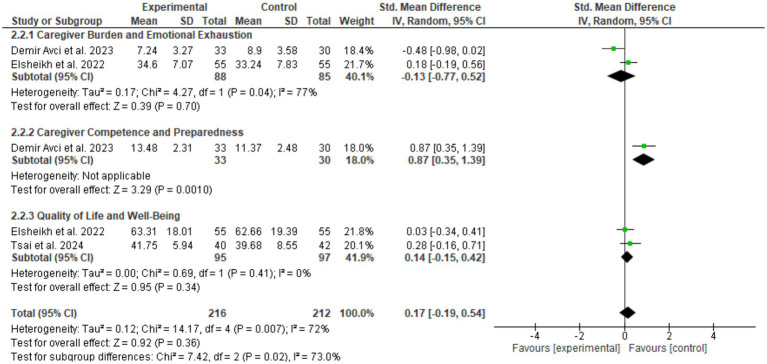
Forest plot of psychosocial outcomes in caregivers, showing SMDs with 95% CI for caregiver burden, emotional exhaustion, competence, preparedness, and quality of life.

Caregiver burden and emotional exhaustion showed no significant improvement (SMD = −0.13, 95% CI: −0.77-0.52, *p* = 0.70), while competence/preparedness improved (SMD = 0.87, 95% CI: 0.35–1.39, *p* = 0.0001). QoL showed limited, non-significant improvement (SMD = 0.14, 95% CI: −0.15-0.42, *p* = 0.34). Sensitivity analyses reduced heterogeneity from 98 to 72% (Figure 6).

### Publication Bias

**Figure 7:**
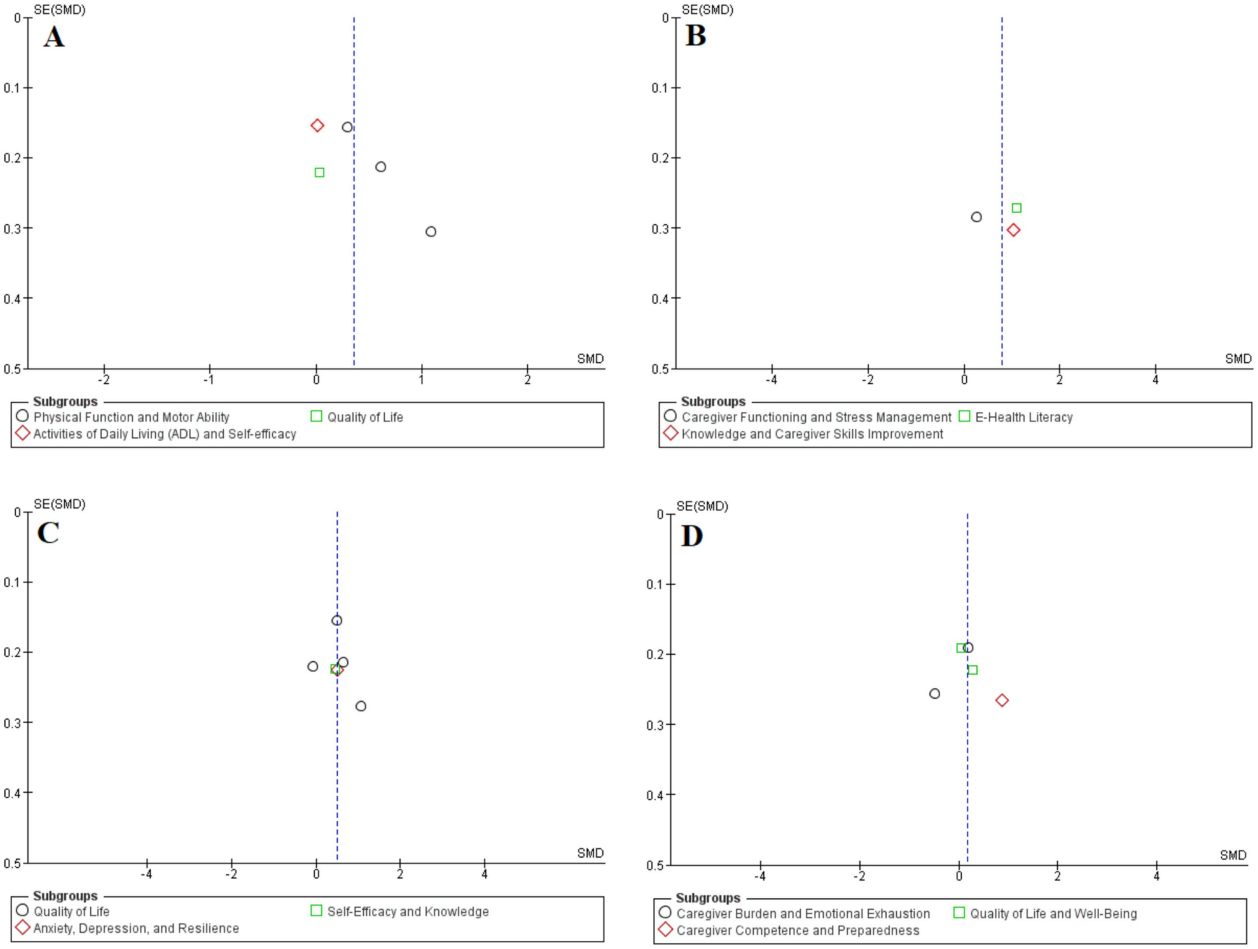
Funnel plots assessing publication bias for functional and psychosocial outcomes among stroke survivors and caregivers: **(A)** physical functioning, activities of daily living, and quality of life; **(B)** caregiver functioning, stress management, e-health literacy, and caregiver skills; **(C)** quality of life, anxiety/depression/resilience, and self-efficacy/knowledge; and **(D)** caregiver burden, competence/preparedness, and quality of life/well-being. The overall distribution of studies appears relatively symmetrical across outcomes.

Funnel plots indicated no significant publication bias across functional and psychosocial outcomes for stroke survivors and caregivers (Figure 7).

### GRADE evidence quality

Table 3 presents moderate to low certainty of evidence on primary outcomes using the GRADE approach. Moderate-quality evidence supports nurse-led caregiver training improving survivors’ physical function/motor ability (SMD 0.60, 95% CI 0.18–1.02), downgraded for serious inconsistency (I^2^ = 65%). Similarly, moderate certainty exists for quality-of-life improvements (SMD 0.51, 95% CI 0.11–0.92; I^2^ = 74%). Low-certainty evidence characterizes all caregiver outcomes knowledge/skills (SMD 1.02, 95% CI 0.42–1.61), e-health literacy (SMD 1.09, 95% CI 0.55–1.62), competence (SMD 0.87, 95% CI 0.35–1.39) and null ADL effects (SMD 0.01, 95% CI -0.29-0.31), primarily due to very serious imprecision from single studies despite large effect estimates. Caregiver burden showed low certainty (SMD -0.13, 95% CI -0.77-0.52; I^2^ = 77%) due to serious inconsistency and imprecision.

**Table 3:** GRADE summary of findings for primary outcomes.

Outcome	Studies (*n*)	Effect size (SMD [95% CI])	Participants (IG/CG)	Certainty of evidence
Survivor physical function/motor	3	0.60 [0.18, 1.02]	152/156	Moderate ⊕ ⊕ ⊕◯
Survivor ADLs/self-efficacy	1	0.01 [−0.29, 0.31]	85/85	Low ⊕ ⊕ ◯◯
Survivor quality of life	4	0.51 [0.11, 0.92]	201/203	Moderate ⊕ ⊕ ⊕◯
Caregiver knowledge/skills	1	1.02 [0.42, 1.61]	25/25	Low ⊕ ⊕ ◯◯
Caregiver E-health literacy	1	1.09 [0.55, 1.62]	33/30	Low ⊕ ⊕ ◯◯
Caregiver competence	1	0.87 [0.35, 1.39]	33/30	Low ⊕ ⊕ ◯◯
Caregiver burden	2	-0.13 [−0.77, 0.52]	88/85	Low ⊕ ⊕ ◯◯

## Discussion

This review synthesized eight RCTs from Taiwan, India, China, Egypt, Turkey, and Iran, evaluating nurse-led caregiver training after stroke in hospital, community, and rehabilitation settings. Interventions, lasting 3–26 weeks with 1–6 months follow-up, were structured, skill-focused, and tailored to caregiver needs, targeting both functional recovery and psychosocial support. Delivery modes included therapeutic conversations, health coaching, home visits, follow-up, and technology-enabled education (videos, laptops, web platforms, WhatsApp). Core components included needs assessments, goal setting, teach-back methods, peer support, and multidimensional care, with comparators typically being routine or brief standard education. Recent updates highlight the central role of neuroscience nurses in stroke care, encompassing collaboration with multidisciplinary teams, coordination across the care continuum, and leadership in patient and caregiver education (32).

Nurse-led programs emphasize individualized, context-sensitive care, involving goal setting, teach-back techniques, psychosocial support, and continuous follow-up. They blend direct instruction with technology-enabled education (videos, web platforms, apps) (14). Telehealth, eHealth platforms, mobile apps, and virtual tools supplement traditional education and follow-up, enhancing accessibility, adherence, and continuity of care (33). Nurses acted as facilitators, educators, coaches, coordinators, and content developers, reflecting the multidimensional role in modern stroke care. Evidence-based interventions spanned acute management protocols to reduce treatment times and post-acute support focusing on education, emotional support, caregiver burden reduction, and psychosocial well-being.

The current review indicates that nurse-led caregiver training significantly improved physical function and motor ability in stroke survivors (SMD = 0.60), but showed no meaningful effect on ADL or self-efficacy. Sensitivity analysis, reducing heterogeneity from 99 to 71%, strengthens reliability. Interventions integrating psychoeducation, skill-building, peer support, and continuous assessment were more effective than single-approach programs, consistent with evidence that multimodal interventions reduce caregiver burden and enhance psychological and social QoL (2).

Notably, these functional improvements have moderate-quality evidence, when interpreted in the context of evidence certainty, indicating that the reported benefits are likely to be reliable despite certain variation among studies. Conversely, the lack of a large effect on ADLs is to be viewed with caution, since prior research studies have also shown no serious impacts of caregiver-mediated interventions on functional independence, which is probably due to the complexity of post-stroke functional recovery and the fact that such a recovery requires more intensive and multidisciplinary rehabilitation instead of an ultimate lack of effect (34, 35).

For psychosocial outcomes in stroke survivors, nurse-led training significantly improved quality of life (SMD = 0.51), reduced anxiety and depression (SMD = 0.49), and yielded modest gains in self-efficacy and knowledge (SMD = 0.45). Sensitivity analyses confirmed heterogeneity reduction from 98 to 56%, adding robustness. Digital integration emphasizes telemedicine and tele-nursing to extend support beyond hospitals, positively impacting patient and caregiver outcomes. Multimodal approaches combining education, psychosocial support, and skill development via in-person and digital methods enhanced caregiver knowledge and coping (36).

In caregivers, nurse-led interventions did not significantly reduce burden or emotional exhaustion (SMD = −0.13) but substantially improved knowledge and caregiving skills (SMD = 1.02) and e-health literacy (SMD = 1.09), with moderate effects on competence and preparedness (SMD = 0.87). Quality of life gains were small and non-significant (SMD = 0.14). Sensitivity analyses reduced heterogeneity, affirming reliability. These findings align with prior studies showing that structured nurse-led education and ongoing support enhance caregiver preparedness and confidence, even if stress reduction may require longer-term, multifaceted interventions (37). Similar outcomes were reported in psychoeducational nurse-led programs supporting stroke survivor-caregiver dyads, showing reductions in caregiver burden and improvements in competence and psychosocial outcomes (38).

Nonetheless, such caregiver-related outcomes have low-certainty evidence, mostly because of small sample sizes and the dependence on individual studies to determine several outcomes. Thus, the size of effect seems to be significant in relation to knowledge and literacy but confidence in such estimates is low and they are to be considered as initial. Similarly, the non-significant change in caregiver burden is to be interpreted with care since prior evidence has shown inconsistent and even conflicting results across studies, and there is great variability that makes it difficult to be certain of the actual change (39).

The lack of significant effects on patient ADLs contrasts with studies reporting more robust functional improvements through multimodal interventions combining physical therapy, occupational therapy, and nursing care rather than caregiver training alone (10, 40). Variability in intervention intensity, patient heterogeneity, and rehabilitation context may explain inconsistent functional recovery outcomes (40). Despite high heterogeneity, nurse-led interventions consistently benefit psychosocial outcomes, reducing caregiver strain and improving family functioning, knowledge, skills, and QoL (41). Burnout reduction showed favorable trends but was not statistically significant, suggesting that longer or more intensive multifaceted programs may be necessary (42, 43). Studies in low- and middle-income countries also report medium-to-large beneficial effects on caregiver burden, depression, and anxiety, highlighting the importance of culturally tailored interventions (44, 45). Overall, nurse-led caregiver training improves functional recovery, psychosocial outcomes, and caregiver knowledge and competence. Digital tools and culturally tailored approaches strengthen these effects, supporting accessibility, equity, and quality of care. Nurse-led education is a critical component in both acute and post-acute stroke phases, fostering patient recovery and caregiver preparedness while highlighting the need for more comprehensive strategies to address caregiver psychosocial health and patient functional independence.

### Limitations

This review has several limitations. First, heterogeneity in intervention content, duration, and delivery methods may have influenced outcome consistency. Second, the limited number of trials reporting on specific domains, such as activities of daily living and caregiver burden, reduces the strength of conclusions in these areas. Third, some of the results were based on individual studies, with broad confidence intervals and low-GRADE certainty levels which make them unable to draw definite conclusions despite large point estimates. There is also a high level of heterogeneity further restricting between-study comparisons, yet sensitivity analyses significantly enhanced consistency. Fourth, variations in measurement tools and outcome definitions across studies restrict comparability and may contribute to variability in effect sizes. Fifth, most studies were conducted in high-resource healthcare settings, limiting generalizability to diverse cultural and healthcare contexts. Finally, publication bias cannot be fully excluded, as studies with null or negative results may have been underrepresented.

## Conclusion

Nurse-led caregiver training interventions significantly improved physical function and motor ability in stroke survivors, with moderate benefits in psychosocial outcomes such as quality of life, anxiety, and depression. These findings are supported by moderate-certainty evidence for key survivor outcomes, indicating a reasonable level of confidence in these effects despite some heterogeneity. No significant effects were observed for activities of daily living, self-efficacy, or caregiver burden, suggesting functional independence and stress management may require longer-term or multimodal strategies. However, these non-significant findings are based on low-certainty evidence and should be interpreted cautiously. In caregivers, nurse-led programs substantially enhanced knowledge, caregiving skills, competence, and e-health literacy, but had limited impact on quality of life and emotional exhaustion. Sensitivity analyses confirmed the robustness of findings, though moderate heterogeneity remains. Overall, nurse-led interventions strengthen survivor recovery and caregiver preparedness while highlighting the need for comprehensive approaches addressing psychosocial well-being and patient functional independence.

### Future recommendations

Future research should focus on longer-term and multimodal rehabilitation strategies combining nurse-led training with physical therapy, occupational therapy, and psychological counseling to enhance functional independence and psychosocial well-being. Given the low certainty of evidence for several outcomes, particularly activities of daily living and caregiver burden, future studies should prioritize larger sample sizes and more rigorous designs to improve precision and confidence in findings. Limited effects on activities of daily living, self-efficacy, and caregiver burden, future interventions should integrate tailored stress management programs, peer support groups, and culturally adapted approaches. Expanding digital health platforms and tele-nursing can further strengthen caregiver competence and e-health literacy, particularly in resource-limited settings. Large-scale, high-quality RCTs with diverse populations and longer follow-up durations are recommended to establish stronger evidence on sustainability and generalizability of outcomes. Multidisciplinary collaboration is essential, embedding nurse-led education within coordinated stroke care pathways to maximize recovery and caregiver preparedness.

## Data Availability

The original contributions presented in the study are included in the article/Supplementary material, further inquiries can be directed to the corresponding author.

## References

[1] Feigin VL Brainin M Norrving B Martins SO Pandian J Lindsay P . World stroke organization: global stroke fact sheet 2025. Int J Stroke. (2025) 20:132–44. doi: 10.1177/17474930241308142, 39635884 PMC11786524

[2] Madu CS Ajibade VM. Acute stroke management and nursing intervention. Cureus. (2025) 17:e86820. doi: 10.7759/CUREUS.86820 40583923 PMC12206066

[3] Zrelak PA Seagraves KB Belagaje S Dusenbury W García JJ Hadidi NN . Nursing’s role in psychosocial health management after a stroke event: a scientific statement from the American Heart Association. Stroke. (2024) 55:e281–94. doi: 10.1161/STR.0000000000000471, 39155870

[4] Veronese M Vellone E Alvaro R Pucciarelli G. The transitional care from hospital to home for stroke survivors and their caregivers: a systematic review. J Vasc Nurs. (2025) 43:86–98. doi: 10.1016/J.JVN.2025.03.002, 40484579

[5] da Costa FM Do Canto DF Felipe LT Rosset I Paskulin LMG. Educational interventions for training caregivers of stroke survivors: a scoping review. Texto & Contexto - Enfermagem. (2024) 33:e20240111. doi: 10.1590/1980-265X-TCE-2024-0111EN

[6] Wan X Pak J Chau C Wu Y Xu L Gong W. Effects of a nurse-led peer support intervention for stroke survivors: protocol for a randomised controlled trial. BMJ Open. (2022) 12:62531. doi: 10.1136/bmjopen-2022-062531, 35688588 PMC9189841

[7] Mangalabarathi N Devi B Chinnathambi K Nagarajan B. Effectiveness of nurse-led stroke rehabilitation on awareness, activities of daily living and coping in stroke patients at a tertiary care hospital in India. Cureus. (2024) 16:e72843. doi: 10.7759/cureus.72843 39618738 PMC11608604

[8] Wang J Zhang Y Chen Y Li M Jin J. Nurse-led motor function rehabilitation program for acute ischemic stroke: a randomized pilot study. J Nurs Res. (2022) 30:E249. doi: 10.1097/JNR.0000000000000529, 36445316

[9] Lin S Xiao LD Chamberlain D. A nurse-led health coaching intervention for stroke survivors and their family caregivers in hospital to home transition care in Chongqing, China: a study protocol for a randomized controlled trial. Trials. (2020) 21:240. doi: 10.1186/S13063-020-4156-Z, 32131876 PMC7057579

[10] Michael NA Mselle LT Tarimo EM Cao Y. The effectiveness of nurse-led transition care on post-discharge outcomes of adult stroke survivors: a systematic review and meta-analysis. Nurs Open. (2025) 12:e70140. doi: 10.1002/NOP2.70140, 40022522 PMC11871394

[11] Bakas T McCarthy MJ Miller EL. Systematic review of the evidence for stroke family caregiver and dyad interventions. Stroke. (2022) 53:2093–102. doi: 10.1161/STROKEAHA.121.034090, 35264010 PMC9133104

[12] Choo WT Jiang Y Chan KGF Ramachandran HJ Teo JYC Seah CWA . Effectiveness of caregiver-mediated exercise interventions on activities of daily living, anxiety and depression post-stroke rehabilitation: a systematic review and meta-analysis. J Adv Nurs. (2022) 78:1870–82. doi: 10.1111/JAN.15239, 35451521

[13] Bui LK Park M Giap TTT. eHealth interventions for the informal caregivers of people with dementia: a systematic review of systematic reviews. Geriatr Nurs. (2022) 48:199–209. doi: 10.1016/J.GERINURSE.2022.09.015 36274510

[14] Zhang W Mei Z Feng Z Li B. Effects of a nurse-led eHealth programme on functional outcomes and quality of life of patients with stroke: a pooled analysis of randomized controlled trials. Front Public Health. (2024) 12:1395270. doi: 10.3389/FPUBH.2024.1395270, 38737865 PMC11082325

[15] McGlinchey MP James J McKevitt C Douiri A Sackley C. The effect of rehabilitation interventions on physical function and immobility-related complications in severe stroke: a systematic review. BMJ Open. (2020) 10:e033642. doi: 10.1136/BMJOPEN-2019-033642, 32029489 PMC7045156

[16] Nungulo VN Gonçalves M Henriques MA Baixinho CL. Effectiveness of psychoeducational intervention in promoting post-stroke self-care: a systematic literature review. Front Rehabil Sci. (2025) 6:1569526. doi: 10.3389/FRESC.2025.1569526, 40547398 PMC12179131

[17] Li C Fang Y Wang H Lin B Xie Y He Y . Correlates of preparedness for caregiving of poststroke patients: a meta-analysis. Front Neurol. (2025) 16:1465962. doi: 10.3389/fneur.2025.1465962, 40510204 PMC12158704

[18] McCurley JL Funes CJ Zale EL Lin A Jacobo M Jacobs JM . Preventing chronic emotional distress in stroke survivors and their informal caregivers. Neurocrit Care. (2019) 30:581. doi: 10.1007/S12028-018-0641-6 30421266 PMC6958510

[19] Bakas T Miller E Sucharew H Kreitzer N Israel J Rota M . Telehealth assessment and skill-building intervention for stroke caregivers (TASK III): study protocol for a randomized controlled clinical trial. JMIR Res Protoc. (2025) 14:e67219. doi: 10.2196/67219 39937971 PMC11979539

[20] Osei SKJ Bempah EA Yeboah AA Owiredu LA Ohene LA. Nurse-led telerehabilitation intervention to improve stroke efficacy: protocol for a pilot randomized feasibility trial. MedRxiv. (2023):2023.01.13.23284509. doi: 10.1101/2023.01.13.23284509 PMC10237469 37267261

[21] Yaqoob E Khan S Sahitia N Barkatullah Z Zaidi DA Khan SA . Caregiver burden in stroke care: identifying predictors and effective interventions – a narrative review. Ann Med Surg. (2025) 87:3458. doi: 10.1097/MS9.0000000000003254, 40486611 PMC12140692

[22] Nejadghaderi SA Balibegloo M Rezaei N. The Cochrane risk of bias assessment tool 2 (RoB 2) versus the original RoB: a perspective on the pros and cons. Health Sci Rep. (2024) 7:e2165. doi: 10.1002/HSR2.2165, 38835932 PMC11147813

[23] Schünemann HJ Mustafa RA Brozek J Steingart KR Leeflang M Murad MH . GRADE guidelines: 21 part 1. Study design, risk of bias, and indirectness in rating the certainty across a body of evidence for test accuracy. J Clin Epidemiol. (2020) 122:129–41. doi: 10.1016/J.JCLINEPI.2019.12.020, 32060007

[24] Tsai SJ Li CC Pai HC. Effects of a nurse-led therapeutic conversations intervention in stroke patient–family caregiver dyads: a randomized control trial. Int J Nurs Pract. (2024) 30:e13257. doi: 10.1111/IJN.13257, 38570203

[25] Devi B. Impact of nurse-led stroke education program (NSEP) on ADL and SS-QOL among patients with stroke and burden among caregivers. Int J Nutr Pharmacol Neurol Dis. (2022) 12:253–62. doi: 10.4103/IJNPND.IJNPND_47_22

[26] Lin S Xiao LD Chamberlain D Ullah S Wang Y Shen Y . Nurse-led health coaching programme to improve hospital-to-home transitional care for stroke survivors: a randomised controlled trial. Patient Educ Couns. (2022) 105:917–25. doi: 10.1016/J.PEC.2021.07.020, 34294494

[27] He Y Yi J Zhu R Zhang J Guo Y Yang Z . Video teach-back training method for family caregivers in stroke continuous rehabilitation: a randomized controlled trial. Geriatr Nurs (Minneap). (2025) 64:103385. doi: 10.1016/J.GERINURSE.2025.103385, 40446752

[28] Elsheikh MA Moriyama M Rahman MM Kako M El-Monshed AH Zoromba M . Effect of a tailored multidimensional intervention on the care burden among family caregivers of stroke survivors: a randomised controlled trial. BMJ Open. (2022) 12:e049741. doi: 10.1136/BMJOPEN-2021-049741 PMC8852666 35168963

[29] Demir Avci Y Gözüm S. Effects of transitional care model-based interventions for stroke patients and caregivers on caregivers’ competence and patient outcomes: randomized controlled trial. Comp Inform Nurs. (2023) 41:805–14. doi: 10.1097/CIN.0000000000000991, 36749850

[30] Urcan Z Kolcu M. Effect of a nurse-led education program for stroke patients on sleep quality and quality of life: a randomized controlled study. Clin Nurs Res. (2022) 31:340–7. doi: 10.1177/10547738211046138, 34519555

[31] Nazari AM Abbaszadeh A Kazemi R Yousofvand V Zandi M. The effect of online training based on stroke educational program on patient’s quality of life and caregiver’s care burden: a randomized controlled trial. BMC Nurs. (2024) 23:958. doi: 10.1186/S12912-024-02629-X, 39736718 PMC11686964

[32] Ashcraft S Wilson SE Nyström KV Dusenbury W Wira CR Burrus TM. Care of the Patient with acute ischemic stroke (prehospital and acute phase of care): update to the 2009 comprehensive nursing care scientific statement: a scientific statement from the American Heart Association. Stroke. (2021) 52:E164–78. doi: 10.1161/STR.0000000000000356, 33691468

[33] Camicia M Lutz B Summers D Klassman L Vaughn S. Nursing’s role in successful stroke care transitions across the continuum: from acute care into the community. Stroke. (2021) 52:E794–805. doi: 10.1161/STROKEAHA.121.033938, 34727736

[34] Chao F Duan X Kang Y Liang N Lu D. Home-based rehabilitation nursing for post-stroke motor dysfunction: an evidence summary. BMC Nurs. (2025) 25:19. doi: 10.1186/S12912-025-04183-6 41345849 PMC12797412

[35] Zhou B Zhang J Zhao Y Li X Anderson CS Xie B . Caregiver-delivered stroke rehabilitation in rural China: the RECOVER randomized controlled trial. Stroke. (2019) 50:1825–30. doi: 10.1161/STROKEAHA.118.021558 31177978

[36] Mondal J Mandal D Bera S Dolai B. Bridging the knowledge gap with multimodal teaching for stroke caregivers. Ukrainian Sci Med Youth J. (2025) 154:190–7. doi: 10.32345/USMYJ.2(154).2025.190-197

[37] Xing X Pu L Hu W Xiao Y Xiao H. The impact of dyadic interventions on psycho-social outcomes for stroke patients and their caregivers: a systematic review and meta-analysis. Front Public Health. (2025) 13:1583621. doi: 10.3389/fpubh.2025.1583621, 40416656 PMC12098548

[38] Bakhtiari-Dovvombaygi H Zare-Kaseb A Nazari AM Rezazadeh Y Bahramnezhad F. The effect of interventions on quality of life, depression, and the burden of Care of Stroke Patients and Their Caregivers: a systematic review. J Neurosci Nurs. (2025) 57:44–50. doi: 10.1097/JNN.0000000000000803, 39514887

[39] Hongwei Y Juan W Hui J Zhongjian L Xuechun L Yaqiong A . Factors influencing the caregiver burden on primary family caregivers of stroke survivors: a scoping review. Qual Life Res. (2026) 35:119. doi: 10.1007/S11136-026-04236-6, 41920479 PMC13043595

[40] Kumar A Kumar M Verma P Pal R Nagi M Mahesh KV . Effects of stroke nurse-led acute stroke management on treatment time benchmarks, intravenous thrombolysis rates, and patient outcomes: a systematic review and meta-analysis. J Stroke Cerebrovasc Dis. (2025) 34:108216. doi: 10.1016/J.JSTROKECEREBROVASDIS.2024.108216, 39740694

[41] Huang H Zhang X Tu L Zhang L Chen H. Effectiveness of nurse-led self-care interventions on quality of life, social support, depression and anxiety among people living with HIV: a systematic review and meta-analysis of randomized controlled trials. Int J Nurs Stud. (2025) 161:104916. doi: 10.1016/J.IJNURSTU.2024.104916 39378740

[42] Sakashita C Endo E Ota E Oku H. Effectiveness of nurse-led transitional care interventions for adult patients discharged from acute care hospitals: a systematic review and meta-analysis. BMC Nurs. (2025) 24:379. doi: 10.1186/S12912-025-03040-W, 40197243 PMC11974112

[43] Caicedo-Fajardo DJ Perdomo-Romero AY Cantillo-Medina CP de Souza ML Ramírez-Perdomo CA. Impact of health interventions on informal caregivers: a systematic review and meta-analysis. Collegian. (2024) 31:437–45. doi: 10.1016/J.COLEGN.2024.10.005

[44] Kasa AS Drury P Traynor V Lee SC Chang HC. The effectiveness of nurse-led interventions to manage frailty in community-dwelling older people: a systematic review. Syst Rev. (2023) 12:182. doi: 10.1186/S13643-023-02335-W, 37777786 PMC10543273

[45] Becqué YN Rietjens JAC van Driel AG van der Heide A Witkamp E. Nursing interventions to support family caregivers in end-of-life care at home: a systematic narrative review. Int J Nurs Stud. (2019) 97:28–39. doi: 10.1016/J.IJNURSTU.2019.04.011, 31132687

