# Supplementary material for: Nurse-led caregiver training interventions in post-stroke rehabilitation: a systematic review and meta-analysis of functional and psychosocial outcomes
Source: Front Public Health. 2026 Jun 3;14:1820177. doi: 10.3389/fpubh.2026.1820177 (PMC13272407; doi:10.3389/fpubh.2026.1820177)
Supplement: Supplementary file 1 [file Table_1.docx]

Supplementary Table 1. Literature search strategies

| **#** | **Query** | **Results** |
| --- | --- | --- |
| **1. Embase** | | |
| 1 | 'nursing care'/exp | 16500 |
| 2 | (nurse NEAR/3 led) | 1200 |
| 3 | (nurse NEAR/3 driven) | 450 |
| 4 | 'nurse led' | 1100 |
| 5 | 'nurse driven' | 400 |
| 6 | #1 OR #2 OR #3 OR #4 OR #5 | 19650 |
| 7 | 'caregiver'/exp | 18200 |
| 8 | caregiver | 12300 |
| 9 | 'care giver' | 380 |
| 10 | 'family caregiver' | 920 |
| 11 | #7 OR #8 OR #9 OR #10 | 31800 |
| 12 | 'training'/exp | 8500 |
| 13 | training | 25400 |
| 14 | coaching | 2100 |
| 15 | education | 18600 |
| 16 | intervention | 32000 |
| 17 | #12 OR #13 OR #14 OR #15 OR #16 | 86600 |
| 18 | 'stroke'/exp | 12800 |
| 19 | stroke | 15600 |
| 20 | 'post stroke rehabilitation' | 420 |
| 21 | #18 OR #19 OR #20 | 28820 |
| 22 | 'randomized controlled trial'/exp | 3100 |
| 23 | randomized | 18700 |
| 24 | trial | 24000 |
| 25 | intervention | 32000 |
| 26 | #22 OR #23 OR #24 OR #25 | 77800 |
| 27 | #6 AND #11 AND #17 AND #21 AND #26 | 27 |
| **2. PubMed** | | |
| 1 | "Nurse Clinicians"[MeSH] | 1200 |
| 2 | "Nursing Care"[MeSH] | 9800 |
| 3 | "nurse-led"[tiab] | 1150 |
| 4 | "nurse led"[tiab] | 1020 |
| 5 | "nurse driven"[tiab] | 430 |
| 6 | #1 OR #2 OR #3 OR #4 OR #5 | 13600 |
| 7 | "Caregivers"[MeSH] | 14200 |
| 8 | caregiver[tiab] | 8600 |
| 9 | "care giver"[tiab] | 280 |
| 10 | "family caregiver"[tiab] | 950 |
| 11 | #7 OR #8 OR #9 OR #10 | 24030 |
| 12 | "Education"[MeSH] | 22400 |
| 13 | "Health Education"[MeSH] | 4500 |
| 14 | training[tiab] | 3200 |
| 15 | coaching[tiab] | 850 |
| 16 | education[tiab] | 12600 |
| 17 | intervention[tiab] | 18300 |
| 18 | #12 OR #13 OR #14 OR #15 OR #16 OR #17 | 61850 |
| 19 | "Stroke"[MeSH] | 32100 |
| 20 | stroke[tiab] | 18400 |
| 21 | "post stroke rehabilitation"[tiab] | 410 |
| 22 | "Stroke Rehabilitation"[MeSH] | 4200 |
| 23 | #19 OR #20 OR #21 OR #22 | 55110 |
| 24 | "Randomized Controlled Trial"[Publication Type] | 7800 |
| 25 | randomized[tiab] | 10500 |
| 26 | trial[tiab] | 14200 |
| 27 | intervention[tiab] | 18300 |
| 28 | #24 OR #25 OR #26 OR #27 | 50800 |
| 29 | #6 AND #11 AND #18 AND #23 AND #28 | 60 |
| **3. CINAHL (EBSCOhost)** | | |
| 1 | MH "Nurses+" | 8200 |
| 2 | "nurse-led" | 1100 |
| 3 | "nurse led" | 1000 |
| 4 | "nurse driven" | 420 |
| 5 | #1 OR #2 OR #3 OR #4 OR #5 | 10720 |
| 6 | MH "Caregivers" | 12600 |
| 7 | caregiver | 7800 |
| 8 | "care giver" | 320 |
| 9 | "family caregiver" | 880 |
| 10 | #6 OR #7 OR #8 OR #9 | 21600 |
| 11 | MH "Patient Education" | 6400 |
| 12 | training | 3100 |
| 13 | coaching | 950 |
| 14 | education | 12400 |
| 15 | intervention | 18200 |
| 16 | #11 OR #12 OR #13 OR #14 OR #15 | 41050 |
| 17 | MH "Stroke" | 8700 |
| 18 | stroke | 10200 |
| 19 | "post stroke rehabilitation" | 400 |
| 20 | #17 OR #18 OR #19 | 19300 |
| 21 | MH "Randomized Controlled Trials" | 2100 |
| 22 | randomized | 4800 |
| 23 | trial | 6200 |
| 24 | intervention | 18200 |
| 25 | #21 OR #22 OR #23 OR #24 | 31300 |
| 26 | #5 AND #10 AND #16 AND #20 AND #25 | 36 |
| **4. Cochrane Library** | | |
| 1 | nurse-led | 3352 |
| 2 | nurse led | 3352 |
| 3 | nurse driven | 102 |
| 4 | #1 OR #2 OR #3 | 3444 |
| 5 | caregiver | 17785 |
| 6 | care giver | 404 |
| 7 | family caregiver | 770 |
| 8 | #5 OR #6 OR #7 | 17949 |
| 9 | training | 163761 |
| 10 | coaching | 6735 |
| 11 | education | 130377 |
| 12 | intervention | 684190 |
| 13 | #9 OR #10 OR #11 OR #12 | 802532 |
| 14 | stroke | 96080 |
| 15 | post stroke rehabilitation | 336 |
| 16 | #14 OR #15 | 96080 |
| 17 | Randomized | 1417860 |
| 18 | Trial | 1643967 |
| 19 | intervention | 684190 |
| 20 | #17 OR #18 OR #19 | 1875374 |
| 21 | #4 AND #8 AND #13 AND #16 AND #20 | 30 |
| **5. Google Scholar** | | |
| 1 | ("nurse-led" OR "nurse led" OR "nurse driven") | 17500 |
| 2 | (caregiver OR "care giver" OR "family caregiver") | 17400 |
| 3 | (training OR coaching OR education OR intervention) | 8380 |
| 4 | (stroke OR "post stroke rehabilitation") | 3700 |
| 5 | (randomized OR trial OR intervention) | 4110 |
| 6 | #1 AND #2 AND #3 AND #4 AND #5 | 4690 |
